# Stable and Lead‐Safe Polyphenol‐Encapsulated Perovskite Solar Cells

**DOI:** 10.1002/advs.202403057

**Published:** 2024-06-18

**Authors:** Shahriyar Safat Dipta, Andrew J. Christofferson, Priyank V. Kumar, Varun Kundi, Muhammad Hanif, Jianbo Tang, Nieves Flores, Kourosh Kalantar‐Zadeh, Ashraf Uddin, Md. Arifur Rahim

**Affiliations:** ^1^ School of Photovoltaic and Renewable Energy Engineering University of New South Wales Sydney New South Wales 2052 Australia; ^2^ School of Science STEM College RMIT University Melbourne Victoria 2476 Australia; ^3^ School of Chemical Engineering University of New South Wales (UNSW) Sydney New South Wales 2052 Australia; ^4^ School of Chemical and Biomolecular Engineering University of Sydney Sydney New South Wales 2006 Australia; ^5^ Department of Chemical and Biological Engineering Monash University Clayton Victoria 3800 Australia

**Keywords:** encapsulation, lead toxicity, Perovskite solar cells, polyphenol coating, stability

## Abstract

Lead (Pb) halide perovskite solar cells (PSCs) exhibit impressive power conversion efficiencies close to those of their silicon counterparts. However, they suffer from moisture instability and Pb safety concerns. Previous studies have endeavoured to address these issues independently, yielding minimal advancements. Here, a general nanoencapsulation platform using natural polyphenols is reported for Pb‐halide PSCs that simultaneously addresses both challenges. The polyphenol‐based encapsulant is solution‐processable, inexpensive (≈1.6 USD m^−2^), and requires only 5 min for the entire process, highlighting its potential scalability. The encapsulated devices with a power conversion efficiency of 20.7% retained up to 80% of their peak performance for 2000 h and up to 70% for 7000 h. Under simulated rainfall conditions, the encapsulant rich in catechol groups captures the Pb ions released from the degraded perovskites via coordination, keeping the Pb levels within the safe drinking water threshold of 15 ppb.

## Graphical abstract

A natural polyphenol‐based nanoencapsulation coating is developed for lead halide perovskite solar cells (PSCs). The encapsulant is low‐cost, scalable, and offers a single, comprehensive solution for enhancing cell stability and safety. The encapsulant effectively stabilizes PSCs in ambient conditions while also safeguarding against lead contamination under severe conditions.

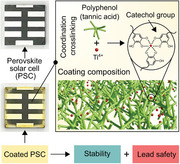

## 1 Introduction

Modern photovoltaics (PVs) for efficiently harnessing sunlight show considerable promise for mitigating fossil fuel dependence and the impending energy crisis. Currently, hybrid Pb‐halide perovskite solar cells (PSCs) are the fastest‐growing PV technology with immense potential, as evidenced by their certified power conversion efficiency (PCE), which has increased to >26% over the past decade.^[^ ^1^, ^2^ ^]^ This rapid progress can be attributed to the exceptional features of Pb‐halide perovskite materials, including bandgap tunabilities, high absorption coefficients, and prolonged carrier lifetimes.^[^ ^2^, ^3^, ^4^ ^]^ In addition, the use of off‐the‐shelf chemicals and solution processabilities enable cost‐effective and scalable PSC device manufacturing.^[^ ^5^, ^6^ ^]^ However, poor stability and potential Pb contamination are two major obstacles for PSCs that limit their commercial prospects.^[^ ^1^, ^2^, ^3^, ^4^, ^7^, ^8^, ^9^, ^10^, ^11^ ^]^ To overcome these challenges, Pb‐free perovskite formulations and various encapsulation strategies have been explored.^[^ ^12^, ^13^, ^14^, ^15^, ^16^, ^17^ ^]^

Although Pb‐free PSCs are a safer option, they also display poor stability, and their efficiency is not competitive with that of Pb‐halide PSCs.^[^ ^12^, ^13^, ^18^ ^]^ Different encapsulation approaches have been attempted to address the challenges of long‐term stability and Pb contamination separately.^[^ ^18^, ^19^, ^20^, ^21^, ^22^, ^23^ ^]^ For example, one strategy involves the use of encapsulating tapes specifically designed for on‐device Pb sequestration; however, this approach has yet to demonstrate long‐term stability.^[^ ^14^ ^]^ On the other hand, the use of internally sandwiched encapsulants has been developed, offering improved stability but failing to address Pb contamination concerns.^[^ ^16^ ^]^ Therefore, the development of a general encapsulation strategy capable of providing a single solution for both long‐term stability and Pb sequestration capacity while remaining synthetically facile and inexpensive is in high demand.

The assembly of natural polyphenols to create conformal coatings is an emerging surface engineering strategy.^[^ ^24^, ^25^, ^26^ ^]^ Polyphenol assembly via coordination cross‐linking is a rapid, versatile, and adaptive method for functionalizing virtually any surface. These coatings also benefit from the diverse physicochemical properties of polyphenols, including universal adhesion, metal coordination, stimulus responsiveness, and redox properties.^[^ ^26^, ^27^ ^]^ For example, pH‐responsive polyphenol coatings have been used as protective encapsulants in living systems, such as yeast and probiotic cells, to enhance their survival under harsh conditions.^[^ ^28^, ^29^ ^]^ Furthermore, the catechol groups present in polyphenols are known to trap hazardous metal ions in polluted water.^[^ ^30^ ^]^ Considering these traits, we hypothesised that polyphenol‐based conformal coatings with appropriate metal–polyphenol combinations could provide a general encapsulation platform for perovskite materials.

**Figure 1:**
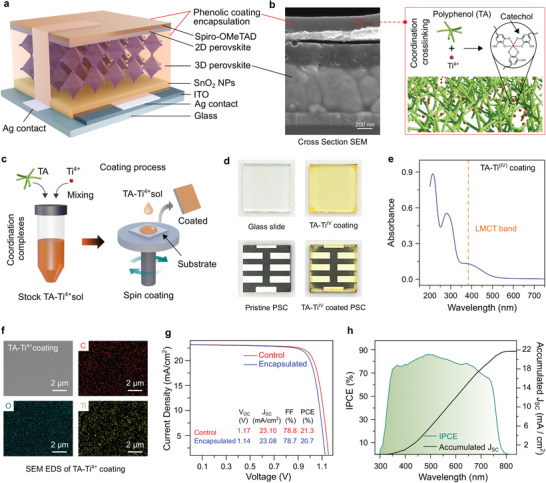
Encapsulated device structure and coating characterisation. a) A 3D schematic illustration of the full device with the polyphenol encapsulant. b) Cross‐section SEM image of the encapsulated device and the molecular view of the encapsulant, crosslinked coordination networks of the TA‐Ti^4+^ coating, where catechol groups are present in TA. c) Schematic of the polyphenol encapsulation process. d) Photographs of the prepared cells on glass substrates with and without encapsulation. e) UV‒vis absorption spectra of the TA‐Ti^4+^ coating showing the LMCT band. f) SEM‒EDS analyses of the TA‐Ti^4+^ coating showing its composition. g) Current density versus voltage performance of the control and encapsulated devices. h) Incident photon‐to‐current conversion efficiency (IPCE) of the encapsulated PSCs in the visible spectrum along with accumulated J_SC_.

In this study, we demonstrate a polyphenol‐based encapsulation strategy for the long‐term stabilisation of PSCs with an inherent capacity to capture Pb ions. A polyphenol‐based coating was deposited on the rear electrode of the PSC device (**Figure** **1**a). For coordination‐driven coating formation, we selected a combination of a natural polyphenol, tannic acid (TA, structure provided in Figure S1, Supporting Information), and titanium ions (Ti^4+^) as the network crosslinking agent (Figure 1b). This coating strategy was developed from a previously reported gel system of TA with group (IV) transition metals (referred to as metal–phenolic gels^[^ ^30^ ^]^). In contrast to other polyphenol‐based coatings with Fe^3+^ or Cu^2+^ via interfacial assembly in aqueous conditions,^[^ ^24^, ^25^, ^26^ ^]^ the metal–phenolic gel system is not solvent‐specific, can incorporate other functional compounds^[^ ^30^ ^]^ and exhibits high mechanical stabilities owing to the strong affinity of the catechol groups of TA for Ti^4+^ ions.^[^ ^26^ ^]^ In addition, by controlling the gelation time, the metal–phenolic sol (initial mix before gelation occurs) can be transformed into thin films via spin coating, as shown in Figure 1c.

## 2 Results and Discussion

### 2.1 Synthesis and Characterization of the PSCs and Encapsulants

The TA‐Ti^4+^ coating on the PSC devices was prepared by mixing the components in methanol. Although TA is soluble in a wide range of solvents, we selected methanol because it can act as an antisolvent for perovskites, and the TA‐Ti^4+^ coating could be successfully prepared without degrading PSC devices. The entire coating process, from compound mixing to spin coating (40 s) and drying, was completed within 5 min. In addition, according to our analysis, the coating would cost (materials) ≈25 and 1.6 USD m^−2^ for laboratory‐scale and large‐scale production, respectively (Note S1, Supporting Information). These results highlight the scalability and low‐cost production of the coatings on an industrial scale.

A 3D schematic view of the device with the TA‐Ti^4+^ encapsulant is shown in Figure 1a. The encapsulating layer covers the entire device surface, except for parts of the electrodes that remained uncoated for photovoltaic data acquisition. The perovskite used in this study has a mixed cation and halide structure with the formula Cs_0.06_Rb_0.04_FA_0.77_MA_0.13_PbI_2.55_Br_0.45_. Details of the perovskite characterisation are presented Figures S2–S5 (Supporting information). The encapsulated device assumes the structure of a glass/ITO/SnO_2_/perovskite/2D passivation layer/spiro‐OMeTAD/Ag/TA‐Ti^4+^ coating. A cross‐sectional scanning electron microscopy (SEM) image of the encapsulated device is shown in Figure 1b. The encapsulating coating layer is clearly observed as the top layer, with a thickness of ≈200 nm. The coating also exhibits a crack‐free homogeneous structure with no pinholes. The thickness of the underlying perovskite layer is ≈500 nm.

A light‐yellow coloured TA‐Ti^4+^ coating is obtained after spin‐coating (Figure 1d). Photographs of the pristine and TA‐Ti^4+^‐coated PSC devices are shown for comparison. We performed UV–vis absorption spectroscopy on the TA‐Ti^4+^ coating on a quartz substrate that exhibited a characteristic ligand‐to‐metal charge (LMCT) transfer band at ≈385 nm (Figure 1e),^[^ ^30^ ^]^ which is in line with the visual colouration of the coating. This also suggests coordination interactions between the catechol/gallol groups of TA and the Ti^4+^ ions, resulting in successful network formation. In addition, the major peaks observed in the Raman spectra of the TA‐Ti^4+^ coating (Figure S6, Supporting Information) are at ≈1356, 1484, and 1604 cm^−1^, which can be attributed to the aromatic ring vibrations of TA in the crosslinked networks.^[^ ^31^ ^]^ Compared with the peaks of the TA powder, these peaks are slightly shifted to lower intensities, confirming supramolecular network formation in the coating.^[^ ^31^ ^]^ The coating thickness is determined to be ≈233 nm when atomic force microscopy (AFM) is performed on a scratched coating section (Figure S7, Supporting Information), which is in line with the thickness observed from the SEM cross‐section (Figure 1b). From the AFM surface topography analysis, the coating was observed to be smooth with a root‐mean‐square roughness of ≈2 nm. For compositional analyses of the coating, X‐ray photoelectron spectroscopy (XPS) and energy‐dispersive X‐ray spectroscopy (EDS) were performed. From the XPS analyses, the C 1s core‐level spectrum displayed characteristic peaks at binding energies (BE) of ≈286.4 and 288.8 eV (Figure S8, Supporting Information), which can be attributed to C═O and C═O groups originating from TA, respectively. In addition, the Ti 2p core‐level spectrum exhibited two peaks at BEs of ≈459 and 465 eV for Ti 2p_3/2_ and Ti 2p_1/2_, respectively, which can be assigned to Ti^4+^ species in the coating networks.^[^ ^30^, ^32^ ^]^ As shown in Figure 1f, the SEM‒EDS analyses of the TA‐Ti^4+^ coating further corroborated the composition of the coating, where well‐distributed C, O, and Ti signals were observed due to TA and metal cross‐linkers.

### 2.2 Performance of the Encapsulated PSCs

Figure 1g and Figure S9 (Supporting Information) illustrates the current density versus voltage (*J–V*) characteristics of the pristine encapsulated and control devices; their corresponding dark *J–V* characteristics are shown in Figure S10 (Supporting Information). The devices retained 96.8% of their peak PCE after encapsulation, demonstrating a minimal effect on the PV performance. The statistical analysis presented in Figure S11 (Supporting Information) shows that this minor drop in the PCE was primarily due to a slightly lower open‐circuit voltage. The encapsulated devices exhibited negligible hysteresis (<2.5%) over 4000 h, as shown in Figure S12 (Supporting Information). We note that light soaking was needed for ≈6 min after polyphenol encapsulation of the PSCs to reach their maximum PCE (Note S3 and Figure S13, Supporting Information). The average IPCE of five randomly selected encapsulated devices is shown in Figure 1h. The short‐circuit current density (J_SC_) from the IPCE is 22.85 mA cm^−2^, which is slightly higher than their average value (22.58 mA cm^−2^) from the J–V statistics presented in Supporting Information Figure S11 (Supporting Information). The high accumulated J_SC_ from the IPCE supports the potential indoor applications of PSCs.^[^ ^33^, ^34^ ^]^

### 2.3 Long‐Term Stability of the Encapsulated PSCs

**Figure 2:**
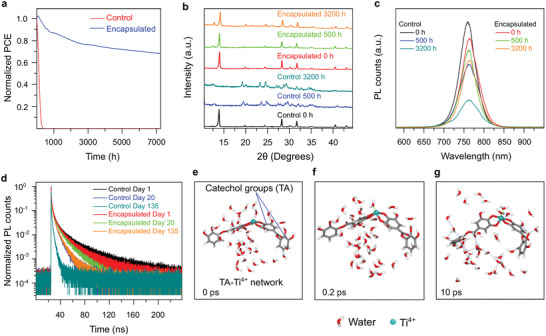
Degradation characteristics of the control and encapsulated devices. a) Normalized PCE of the control and encapsulated PSCs up to 7200 h of the stability test under indoor light at 22 °C and 65% relative humidity. b) The PL spectra of freshly prepared and aged PSCs. c) TrPL spectra of freshly prepared aged devices with and without encapsulation. d) Normalized XRD pattern of the control and encapsulated devices showing a negligible change in peak intensity after 3200 h for the encapsulated device. e–g) Molecular dynamics simulation illustrating the interaction of water molecules with the TA‐Ti^4+^ network in the encapsulant. Initially (at 0 ps), the water molecules in contact with the network start to interact with the TA/Ti^4+^ coordination complexes, and the process of forming stable bonds initiates at the Ti^4+^ center, as shown for 0.2 ps. At 10 ps, water molecules form stable clusters with the network with a net negative energy.

The degradation study of the control and encapsulated devices was performed under indoor lights at 22 °C and 65% relative humidity. The degradation analysis presented in **Figure** **2**a shows that the control device fully degraded within 250 h, whereas the encapsulated devices retained more than 90% (T90) of their initial PCE after 650 h. Furthermore, the encapsulated devices retained 85% (T85), 80% (T80), and 70% (T70) of their initial PCE for more than 1400, 2050, and 7000 h, respectively. A detailed degradation analysis of the cells is presented in Table S1 (Supporting Information). Recently, Xu et al.^[^ ^16^ ^]^ and Wang et al.^[^ ^22^ ^]^ employed conjugation chemistry‐based internal encapsulation and self‐crosslinked fluorosilicone polymer encapsulation strategies, respectively, for PSCs to achieve a T90 lifetime of ≈950 h, which is higher than ours. We note the compositional variation in the PSCs used in our investigations, which were intrinsically less stable. Their control devices showed a T50 lifetime of ≈450 h, whereas our study exhibited a T50 lifetime of only 150 h. Furthermore, these encapsulation strategies are time‐consuming, involve complex synthesis steps, and lack Pb‐absorbing capacities.

The optoelectronic stability of the perovskite film underneath the encapsulant was further probed by photoluminescence (PL) and time‐resolved PL, as illustrated in Figure 2b,c, respectively. Considerable luminescence degradation was observed for the aged control devices, whereas the encapsulated cells showed stronger retention. The PL peaks were observed to shift for both the control and encapsulated devices. For the control device, the redshift of the materials caused by degradation decreased the bandgap within 500 h as shown in Figure 2b.^[^ ^35^ ^]^ The blueshift due to phase segregation for the control devices was not observed because of rapid degradation. For the encapsulated devices, both redshift and blueshift (phase segregation) contributed to the PL peaks. Initially, distinct iodide‐rich and bromide‐rich regions are formed in the thin film due to phase segregation, shifting the PL peak slightly based on the perovskite's halide composition.^[^ ^36^ ^]^ Up to 500 h, the phase segregation was dominant, increasing the bandgap. However, at 3200 h, redshift due to degradation became dominant as evident from the peak shifted to a longer wavelength. Carrier lifetime and decay analyses are listed in Table S2 (Supporting Information) and show a smaller change in carrier lifetime of the encapsulated devices from 2.4 to 1.8 ns after 3200 h. In contrast, the control devices show a significant decline in lifetime from 2.5 to 0.3 ns. In addition, a slight redshift was observed in the PL spectra of the control samples as they degraded, which was not detected in the encapsulated samples for up to 3200 h.

The crystal qualities of the perovskite materials were further analysed by X‐ray diffraction (XRD), as depicted in Figure 2d. The characteristic XRD peaks at 14.1° (100), 28.3° (200), and 31.7° (210) of the perovskite crystals remained almost unaltered even after 3200 h for the encapsulated devices kept in a dark environment.^[^ ^37^ ^]^ In contrast, the peaks of the control device disappeared after 500 h, leaving only the substrate peaks. The retention of nearly pristine quality crystals in the aged encapsulated device further confirms the moisture protection of the perovskite film by the TA‐Ti^4+^ encapsulant.

### 2.4 Inhibition of Water Permeation

Water‐soluble polyphenols interact with water via hydrogen bonding and are typically considered hydrophilic. Therefore, the encapsulant TA‐Ti^4+^ coating presented here is expected to be hydrophilic and may not be considered an ideal encapsulant material. However, our results demonstrate the excellent encapsulation performance of this coating for PSCs. Molecular dynamics simulations were performed to understand this behavior.

First, we formed a coordination network for the coating composed of TA and Ti^4+^ ions (Figure 2e). Next, 40 water molecules were added to interact with the coating network. The calculated interaction energy was −0.27 eV per water molecule (Note S2 and Figure S14, Supporting Information). The negative interaction energy between water and the TA‐Ti^4+^ complex indicated that the binding of water molecules to the complex was energetically favourable. The total energy of the system, which is composed of water molecules and the TA‐Ti^4+^ network, decreases when water molecules form bonds with the TA‐Ti^4+^ complexes in the network. The negative interaction energy value suggests that the bond between water and the TA‐Ti^4+^ complex is energetically stable and that the water molecules exhibit a strong affinity for the complexes. All periodic systems studied in this study are shown in Figure 2e–g. Analysis of the configurations of water molecules interacting with the TA‐Ti^4+^ complex revealed that water molecules formed a cluster around the Ti^4+^ centre (see time points 0.2 and 10 ps, Figure 2e–g). This was achieved by water molecules directly interacting with the Ti^4+^ centre and forming hydrogen bonds with the hydroxyl groups of TA, which led to the formation of water clusters in the coating network (Figure 2g). Overall, these results (Figure 2e–g) suggest that water molecules strongly interact with the coating by forming stable clusters. Therefore, the water molecules cannot penetrate to reach the underlying layers and protect the perovskite layers from degradation. These results suggest that our coating's ability to prevent water permeation is fundamentally different from that of other encapsulating materials that primarily rely on hydrophobic compounds.^[^ ^15^, ^16^, ^20^, ^22^ ^]^

### 2.5 Lead Sequestration by the Encapsulant

**Figure 3:**
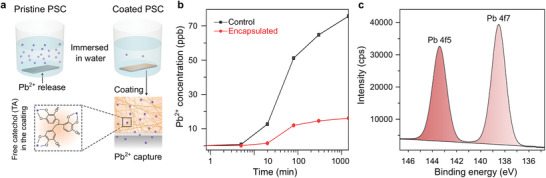
Lead capturing ability of the polyphenol encapsulant. a) Schematic showing capture of Pb^2+^ in the TA‐Ti^4+^ coating when the samples were encapsulated. b) Concentration of Pb^2+^ in water using IPC‐MS after the devices were immersed for up to 24 h. c) Captured Pb^2+^ detected from XPS after the TA‐Ti^4+^ encapsulant was immersed in water for 24 h.

Lead contamination is a significant environmental hazard that poses a serious threat to human health. As such, the use of Pb‐based PSCs in natural environments, such as during rainfall, for practical implementation remains a key concern owing to Pb leaching from the devices.^[^ ^9^ ^]^ As previously mentioned, the TA‐Ti^4+^ coating serves two purposes, namely, to protect the perovskites from degradation in extreme scenarios, such as the PSCs being submerged in water, and the environment from Pb contamination. To illustrate the latter, the control and encapsulated PSCs were immersed in water (**Figure** **3**a), and Pb leaching into the water was monitored over time. Inductively coupled plasma‒mass spectrometry was performed for the water samples collected at different time points after the immersion of the cells. The capture of Pb^2+^ ions^[^ ^29^ ^]^ by the free catechol groups of the TA‐Ti^4+^ coating is schematically shown in Figure 3a. In addition, Figure 3b shows the concentration of Pb^2+^ in water as a function of time. As is evident from these results, the TA‐Ti^4+^ coating was able to capture more than 75% of the Pb^2+^ ions compared with the control samples. The average initial Pb^2+^ leaching rates were 0.043 and 0.0096 ppb min^−1^ for the control and encapsulated samples, respectively. However, after 300 min, the encapsulated samples exhibited significantly reduced Pb^2+^ leaching at a rate of 0.00014 ppb min^−1^, whereas the control samples continued to leach at a rate of 0.001 ppb min^−1^. This indicates that the coating captured more than 90% of the leached Pb^2+^ ions after 300 min. Our calculations show that after a 1.44 cm^2^ encapsulated cell was submerged in 1 L water for 1 day, the Pb content of the water was 10.08 µg L^−1^, which is below the 15 µg L^−1^ limit for drinking water.^[^ ^38^ ^]^ Considering that the perovskite film in the PSC device (1.44 cm^2^) was composed of 33.5% Pb^2+^, the leaching rate could be effectively controlled by the TA‐Ti^4+^ coating. As shown by Li et al.,^[^ ^20^ ^]^ the Pb sequestration capability of a coating containing phosphonic acid groups was ≈96%, which is higher than that of the present TA‐Ti^4+^ coating. However, the coating thickness in that study was ≈2 µm, which is ten times higher than that of our study. Additionally, devices encapsulated with the TA‐Ti^4+^ coating displayed four times higher stability (>80% of the initial PCE) than the encapsulated devices shown in that study.

To further confirm the above observation, we also immersed a coated TA‐Ti^4+^ sample (without perovskites) in a Pb^2+^‐containing solution. After 24 h, the sample was removed, washed three times and dried. The XPS performed on this sample clearly showed Pb^2+^ ions captured in the coating network (Figure S15, Supporting Information). According to the core‐level XPS spectra, two Pb 4f peaks were detected at 138.5 (4f_7/2_) and 143.3 (4f_5/2_) eV, with a spin‐orbit separation of 4.8 eV, suggesting that Pb in the coating existed in a divalent state, as illustrated in Figure 3c. Additionally, the peak (4f_7/2_) shifts to a higher BE in the coating relative to PbO at 136.8 eV (4f_7/2_) and Pb^2+^ in PbO at 137.8 eV,^[^ ^39^, ^40^ ^]^ which could be attributed to the coordination of Pb^2+^ ions with the free catechol groups of TA in the encapsulant.

## 3 Conclusion

In summary, we report a phenolic encapsulant based on a natural polyphenol for perovskite solar cells that greatly improves their lifetime and prevents Pb^2+^ from leaching into the surrounding environment. While the encapsulant is low‐cost and processing‐friendly, the cells retain 96.8% of their PCE after encapsulation. The encapsulated cells retained ≈68.2% of their initial PCE (20.7%) even after 300 days in ambient conditions. XRD analysis confirms that the crystallinity is not degraded in the encapsulated cells. The encapsulated cells show very similar stability to the ones kept in an ideal nitrogen environment, which suggests the encapsulant inhibits external degradation. Computational analysis shows how water molecules interact with the TA‐Ti^4+^ complex to prevent it from passing through the coating, even though the coating is hydrophilic in nature. Mimicking a rainfall scenario, we also demonstrate that the TA‐Ti^4+^ coating can capture more than 80% of the Pb^2+^ ions that would leach out to the surrounding in the absence of the coating. This unveils the opportunity of further research with phenolic materials to resolve all major challenges of PSCs that hinder their commercialization.

## Conflict of Interest

Polyphenol coatings used as encapsulants for the perovskite solar cell have been filed as a patent (application number 2023902538) by the University of New South Wales, Sydney (UNSW Sydney).

## Author Contributions

M.A.R., S.S.D, and A.U performed conceptualization. SSD and MAR performed methodology. S.S.D, M.A.R, M.H, N.F, A.U, A.C, P.K, V.K, J.T, and K.K performed investigation. S.S.D, M.A.R, M.H, A.U, A.C, P.K, V.K, and J.T performed visualization. M.A.R and A.U performed funding acquisition. M.A.R and A.U performed Project administration. M.A.R and A.U performed supervision. M.A.R, S.S.D, A.U A.C, P.K, V.K, J.T and K.K wrote the original draft. M.A.R, SS.D, A.U A.C, P.K, V.K, J.T, and K.K reviewed and edited the final manuscript.

## Supporting information

Supporting Information

## Data Availability

The data that support the findings of this study are available from the corresponding author upon reasonable request.
