# Supplementary material for: Stable and Lead‐Safe Polyphenol‐Encapsulated Perovskite Solar Cells
Source: Adv Sci (Weinh). 2024 Jun 18;11(31):2403057. doi: 10.1002/advs.202403057 (PMC11336907; doi:10.1002/advs.202403057)
Supplement: Supplementary file 1 — Supporting Information [file ADVS-11-2403057-s001.pdf]

ADVANCED SCIENCE
Open Access

Supporting Information

for *Adv. Sci.*, DOI 10.1002/advs.202403057

Stable and Lead-Safe Polyphenol-Encapsulated Perovskite Solar Cells

Shahriyar Safat Dipta, Andrew J. Christofferson, Priyank V. Kumar, Varun Kundi, Muhammad Hanif, Jianbo Tang, Nieves Flores, Kourosh Kalantar-Zadeh, Ashraf Uddin and Md. Arifur Rahim**

Supporting Information

Stable and lead-safe polyphenol-encapsulated perovskite solar cells

Shahriyar Safat Dipta, Andrew J. Christofferson, Priyank V. Kumar, Varun Kundi, Muhammad Hanif, Jianbo Tang, Nieves Flores, Kourosh Kalantar-Zadeh, Ashraf Uddin, Md. Arifur Rahim**

The file includes:

Materials and methods
Supporting text and notes
Supporting Information Figure 1–15
Supplementary Information Table 1 and 2

Materials

The ITO substrates and Spiro-OMeTAD were purchased from Luminescence Technology Corp. (Lumtec). All solvents (except methanol from ChemSupply), caesium iodide (CsI), rubidium iodide (RbI), tannic acid (TA) and titanium(IV) bis(ammonium lactato)dihydroxide solution (50 wt% in H_2O; Ti-BALDH) were purchased from Sigma Aldrich and used as received. Perovskite precursors formamidinium iodide (FAI) and methylammonium bromide (MABr) were purchased from GreatCell Solar Materials. SnO_2 15% dispersion solution in H_2O was purchased from Thermo Fisher Scientific. Lead iodide (PbI_2) and lead bromide ($PbBr_2$) were purchased from TCI. Silver shots were bought from ESPI Metals.

Methods:

Perovskite Solar Cell Fabrication

Patterned ITO on glass substrates (12 cm × 12 cm) were cleaned sequentially with Hellmanex III, deionized water, acetone, and 2-propanol in an ultrasonic bath for 10 minutes in each step. The cleaned substrates were then heated at 70 °C for 30 minutes and treated with UV-ozone for an additional 20 minutes. For the electron transport layer, a 3.75% SnO_2 nanoparticle dispersion solution in H_2O was stirred for 1 h and spin-coated at 3500 rpm for 35 s on clean ITO substrates. The sample was transferred to an open-air hot plate and annealed for 30 min at 150 °C to form a mesoporous SnO_2 layer. The substrates were then placed inside the glove box for perovskite deposition.

$Cs_{0.06}Rb_{0.04}FA_{0.77}MA_{0.13}PbI_{2.55}Br_{0.45}$ perovskite solution (1.3 M) was prepared by mixing solutions from three different bottles (B1, B2, B3). In B1 189.2 mg FAI, 22.4 mg MABr, 553.2 mg PbI_2, and 73.4 mg $PbBr_2$ were dissolved in a 1 mL solution of DMF and DMSO at a ratio of 4:1. In B2, 169 mg of CsI was dissolved in 500 mL DMSO, while in B3, 138 mg RbI was dissolved in 500 mL DMSO. After rigorous stirring for 2 h, 70 mL of solution from B2 and 30 mL from B3 were transferred to B1 to make the final perovskite solution, which was further stirred for 1 h. The solution was spin-coated on top of the SnO_2 layer at a rate of 1000 rpm for 10 s, followed by 4000 rpm for 40 s. The perovskite film was immediately annealed at 110 °C for 10 minutes.

A 72.3 mg Spiro-OMeTAD solution was prepared in 1 mL chlorobenzene. To increase the conductivity, 28.8 μL 4-tBP and 17.5 μL Li-TFSI salt were added and rigorously stirred for 2 h. The solution was then spin-coated on the perovskite thin film at a rate of 3500 rpm for 30 s.

Finally, the electrodes of the device were thermally evaporated using a shadow mask at a rate of 0.1 nm/sec under 10^{-5} m-bar pressure. The electrodes were 100 nm thick. For the first 10 nm, the rate was maintained at 0.01 nm/sec. The cells had an active area of 0.045 cm^2 each. The devices were scratched at all sides so that the encapsulant could cover the edges.

Preparation of the TA–Ti^{4+} coating as the encapsulant layer

In a typical process, TA solution (200 μL, 25 wt.% in methanol, 250 mg mL^{-1}) and Ti^{4+} precursor solution (Ti-BALDH; 50 μL, 50 wt.% aqueous solution) were mixed in a 1.5-mL tube under vortex mixing to achieve a TA/Ti^{4+} molar ratio of ~1:1.4. This mixture (orange–red sol) was immediately diluted (under vortex) with 400 μL of methanol to avoid rapid bulk gelation. A volume of 50 μL from the resulting solution was immediately (within a min) spread over a complete perovskite device (1.44 cm^2), and the device was spin-coated at 5000 rpm for 30 s, followed by annealing at 120 °C for 3 min.

Device and materials characterisations

The photovoltaic (J-V) characterisations were performed with an NREL calibrated Keithley 2400 Source Meter under 100 mW/cm^2 (AM 1.5G) simulated sunlight. X-ray diffraction (XRD) with CuKα radiation was performed by step-scanning with a step size of 0.01 degrees. A Nano SEM 450 field-emission scanning electron microscope (FE-SEM) with a Bruker SDD-EDS detector was used to capture surface topology and device cross-sectional images. The surface roughness and thickness of the encapsulant coating were measured with Bruker Dimension ICON SPM Atomic Force Microscopy (AFM) with a scan size of "1 × 1 μm" at a scan rate of 0.512 Hz. External quantum efficiency (EQE) measurements were performed using a QEX10 spectral response system from PV Measurements Inc. Photoluminescence (PL) characterisations were performed using a pulsed OPO laser at 328 nm with a glacier XTE-cooled CCD spectrometer having a detection range of 200 - 1050 nm. Inductively coupled plasma–mass spectrometry (ICP–MS) analysis was performed using a NexION 5000 Multi-Quadrupole ICP Mass Spectrometer. A Thermo ESCALAB250i high-resolution X-ray photoelectron spectrometer (XPS) was used to perform XPS analysis of the encapsulant and the perovskite layer.

For the TA-Ti^{4+} coating on the PSC devices, a spin-coater (Laurell – Model WS400M-6NPP) was used in a single step with the following parameters: 5000 rpm, 2000 rpm acceleration, and 30 s spin time. UV–Vis absorption spectra were recorded on a Cary 5000 (PerkinElmer) spectrophotometer using a solid-state accessory. The coating was prepared on a quartz substrate to collect the spectra. Raman spectroscopy (inVia Raman microscope, RENISHAW, 532 nm laser source) was performed for coatings deposited on silicon wafers.

Supporting Text

Carrier Lifetime

The bi-exponential model as expressed in Equation S1, was used to extract the PL lifetime from the TrPL decay measurements using Decay Fit software.[1] In Equation S1, τ_1 and τ_2 are the fast and slow decay component, while A_1 and A_2 are the weighting factor of the fast and slow decay component respectively.

$$A = A_1 e^{-\frac{t}{\tau_1}} + A_2 e^{-\frac{t}{\tau_2}} \qquad \text{(Equation S1)}$$

Hysteresis Index

PSCs show hysteresis behaviour depending on the direction of the voltage sweep. The reported J-V figures and data are based on the reverse sweep. However, J-V was measured with sweeping in both directions to find hysteresis of the device as shown in **Figure S9**. Generally, the PCE in the forward sweep is lower than the PCE in the reverse sweep.[2] Hysteresis index is represented by several ways in different works.[3,4] Here, we have used the formula used by Severin *et al.*[4] shown in Equation S2.

$$Hysteresis\ index = \frac{PCE(reverse) - PCE(forward)}{PCE(reverse)} \qquad \text{(Equation S2)}$$

Furthermore, an interesting behaviour was seen in the encapsulated devices the first time the J-V data was measured. The devices need to be kept in the light for some time before they yield their highest current density. The current in the cells builds up from zero to the J_{SC} reported after ~6 mins. **Figure S13** shows the J_{SC} vs Time analysis for a randomly selected encapsulated device. It is notable that this behaviour is only shown when J-V analysis is done first time after encapsulating. In subsequent analysis, this behaviour is not shown.

Materials cost analysis of the phenolic encapsulant (Note 1)

Laboratory scale:

Materials

Tannic acid: 1 kg (Sigma Aldrich): 181 AUD ≅ 117.65 USD

Ti BALDH (50% in H_2O), 500 mL (Sigma Aldrich): 150 AUD ≅ 97.5 USD

Methanol, 500 mL (ChemSupply): 25 AUD ≅ 16.25 USD

For 10 devices,

600 µL methanol = 600 ÷ (1000 × 500) × 25 AUD = 3 cents ≅ 2 cents (US)

50 mg TA = 50 ÷ (1000 ×1000) × 181 AUD = 0.905 cents ≅ 0.6 cents (US)

50 µL of Ti BALDH = 50 ÷ (1000 × 500) × 150 AUD = 1.5 cents ≅ 1 cent (US)

Total Cost:

Total per sample (1.44 square cm) = (3 + 0.905 + 1.5) ÷ 10 cents = 0.54 cents ≅ 0.36 cents (US)

Total material cost per square meter = 25.11 USD

Large scale

Materials

These are based on quotes received from industrial suppliers by the authors

Tannic acid: 1 ton: 181 AUD ≅ 11.6 USD/kg

Ti BALDH 1000 L (50% in H_2O): 12 USD/L

Methanol 1 ton: ≅ 0.55 USD/kg

For 10 devices,

600 µL methanol = 600 ÷ (1000 × 500) × 0.792 × 0.55 USD = 0.052 cents

50 mg TA = 50 ÷ (1000 ×1000) × 11.6 USD = 0.058 cents

50 µL of Ti BALDH = 50 ÷ (1000 × 500) × 12 = 0.12 cents

Total Cost:

Total per sample (1.44 square cm) = (0.052 + 0.058 + 0.12) ÷ 10 cents = 0.023 cents

Total material cost per square meter = 1.59 USD

Payback period calculation

Average efficiency over a 6-month cycle = 20% × 0.85 = 17%

Average peak sun hours per day (Sydney) = 7.1*

*Source: Renogy Australia

Energy harvested per day from a module of 1 m^2 = 1.2 kWh

Market cost of 1.2 kWh = 25.19 ÷ 100 × 1.2 AUD = 0.3 AUD ≅ 0.195 USD

Payback period for the coating = 8.15 days

Molecular dynamics simulation (Note 2)

The molecular dynamics (MD) simulations were carried out using the Vienna Ab-initio Simulation Package (VASP)[5-8] code on a system consisting of 40 water molecules surrounding a Ti-complex, 40 water molecules and Ti-complex separately as well. The cell size for all the simulations was defined as 15 Å for all three cell vectors.

The projector augmented wave (PAW) method[9,10] with PBE exchange-correlation function was employed to describe the ion-electron interactions. The plane wave basis set with a kinetic energy cut-off of 450 eV was used to describe the wave functions. The initial charge density was generated from the atomic positions and spin polarization was not included in the simulation. The real-space projection was set to automatic, and the electronic optimization was performed using algorithm 48.

The sigma value of 0.1 eV and a gamma-point k-grid were used for all the calculations. The simulation was run for a maximum of 10000 ionic steps with a timestep of 1 fs. The simulation was performed in the canonical ensemble with an initial temperature of 300 K and a final temperature of 300 K. The symmetry was switched off, and the output was written every ionic step to monitor the progress of the simulation. The interaction energy of water surrounding the Ti-complex was calculated using Equation S3.

$$E_{interaction} = \langle E_{Ti-Complex+40\,waters} \rangle - \left(\langle E_{Ti-Complex} \rangle + \langle E_{40\,waters} \rangle \right) \quad \text{(Equation S3)}$$

Where $\langle E_{Ti-Complex+40\,waters} \rangle$, $\langle E_{Ti-Complex} \rangle$, and $\langle E_{40\,waters} \rangle$ are the SCF energies averaged over the last 5 ps of the each simulation. The calculated interaction energy using the relationship mentioned above comes out to be -10.71 eV.

Current buildup in the encapsulated devices (Note 3)

During the first I-V measurements (not in the latter measurements), the encapsulated devices show a gradual buildup of current as shown in Figure S13. Though the underlying reason is not well understood, one possible reason is the trapping of solvent molecules in the interfaces of the encapsulant with Ag and Spiro-OMeTAD. These trapped molecules hinder the current flow at the beginning. However, they soon escape the interface following exposure to light and current flows in the device in a regular manner. This phenomenon was only observed in the encapsulated devices in their first time I-V measurement. In the subsequent measurements, no such buildup phase was not observed.

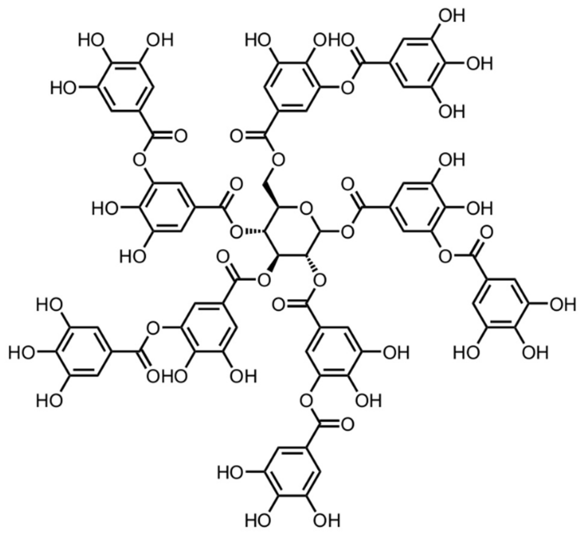

Figure S1. Chemical structure of tannic Acid (TA).

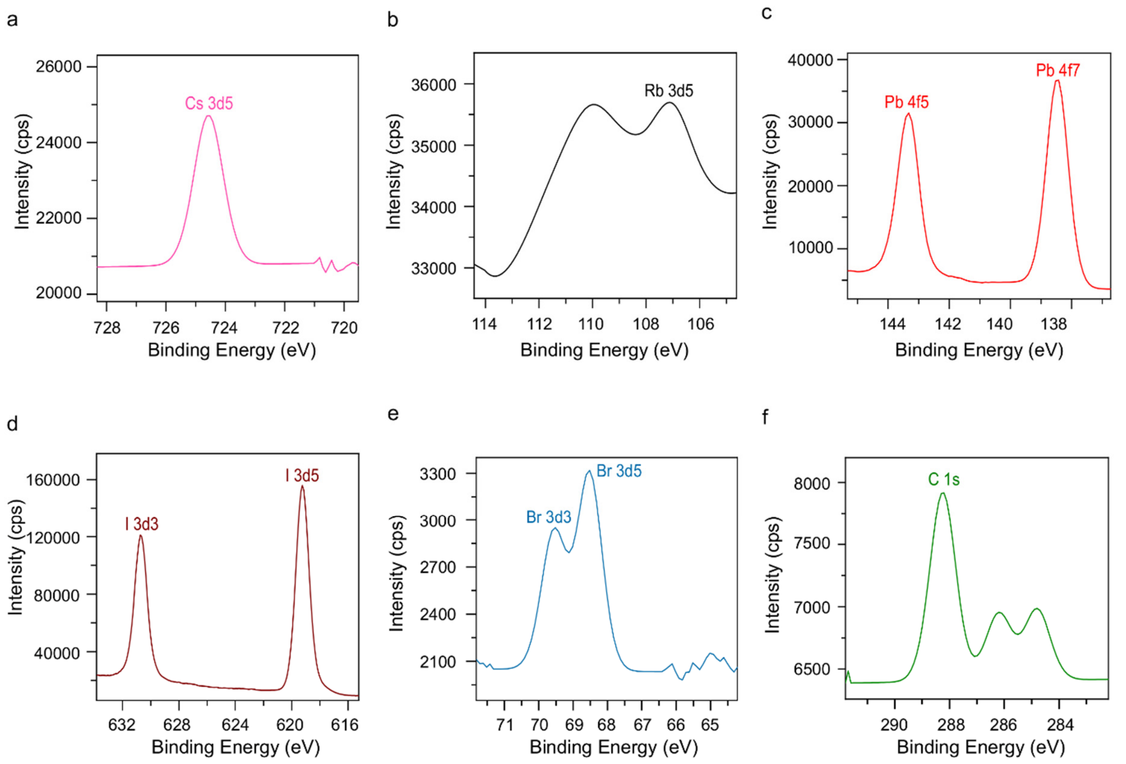

Figure S2. The XPS characterization of the perovskite layer, showing the peaks for (a) Cs, (b) Rb, (c) Pb, (d) I, (e) Br, and (f) C. These elements are in line with the composition of the perovskites used in this study ($Cs_{0.06}Rb_{0.04}FA_{0.77}MA_{0.13}PbI_{2.55}Br_{0.45}$).

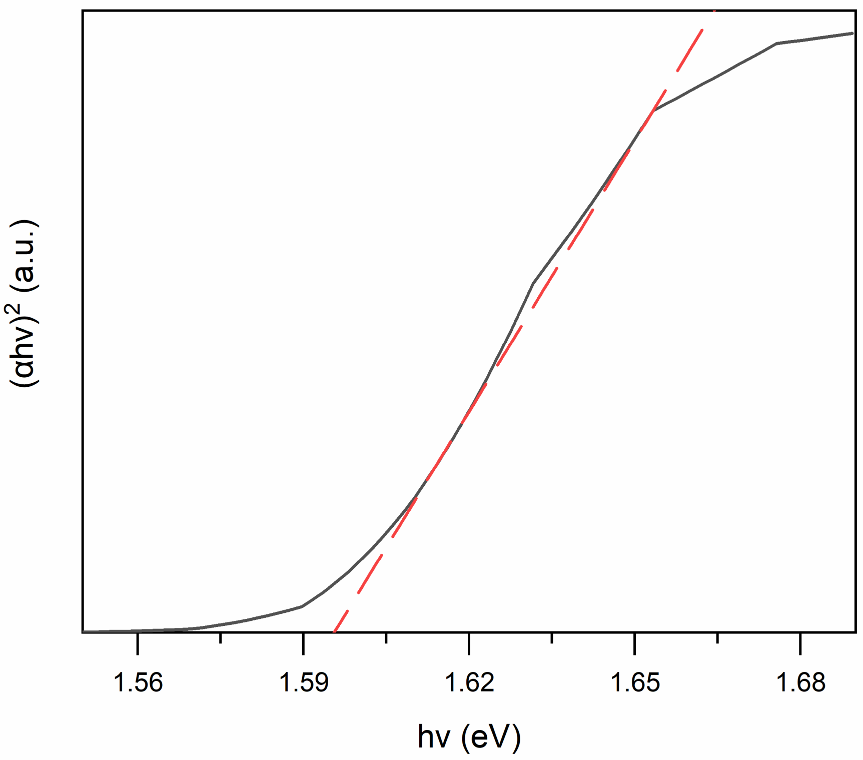

Figure S3. The Tauc plot of a randomly selected encapsulated cell. The bandgap calculated from the plot is ~1.6 eV.

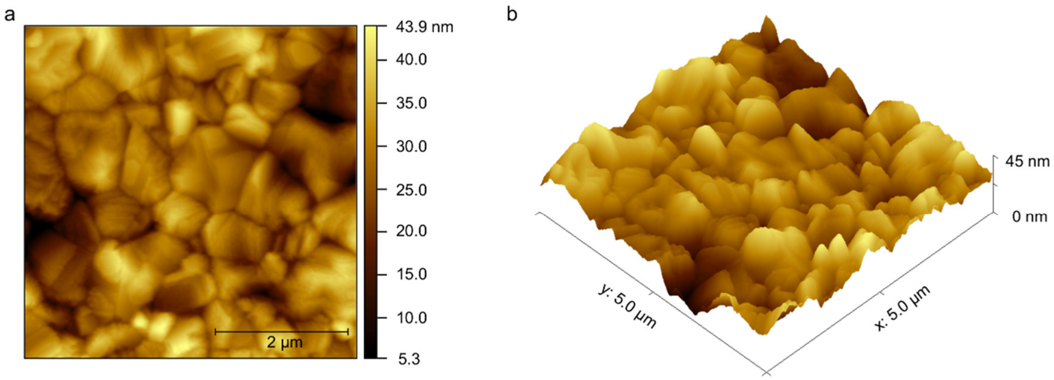

Figure S4. (a) The 2D and (b) 3D view of the surface topology of the perovskite film before encapsulation measured by AFM. The spot size was 5 μm. The RMS roughness of the perovskite was found to be 9.3 nm.

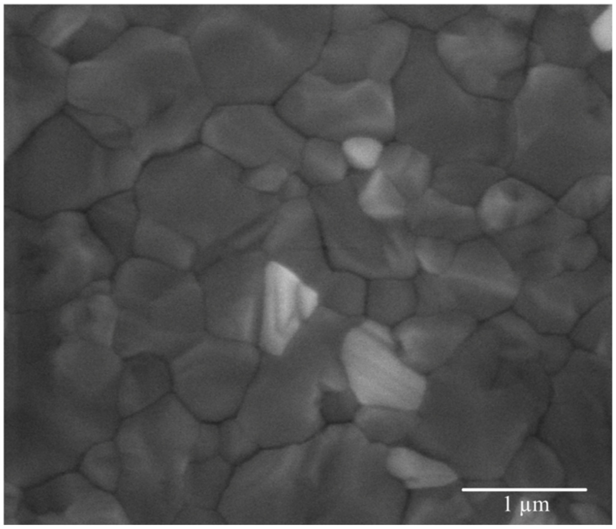

Figure S5. The SEM image the perovskite layer showing its surface morphology.

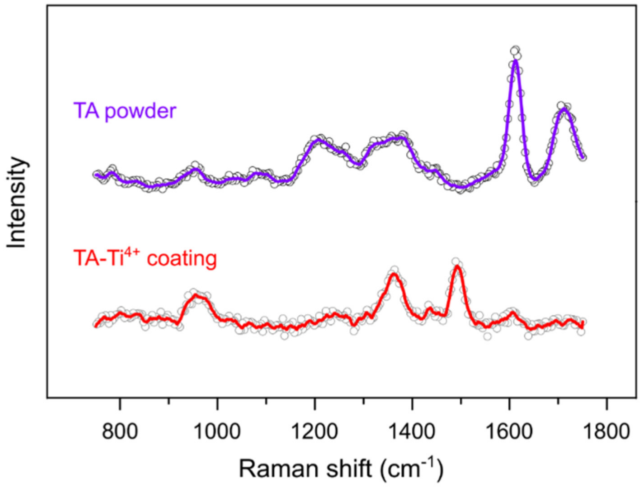

Figure S6. Raman spectra of the TA–Ti^{4+} coating and TA powder.

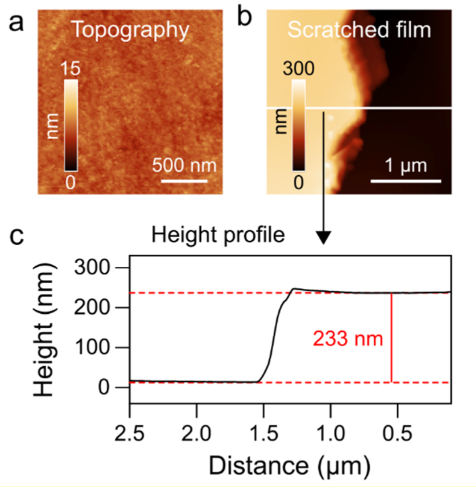

Figure S7. AFM surface topography (a) and thickness analyses (b,c) of the TA–Ti^{4+} coating.

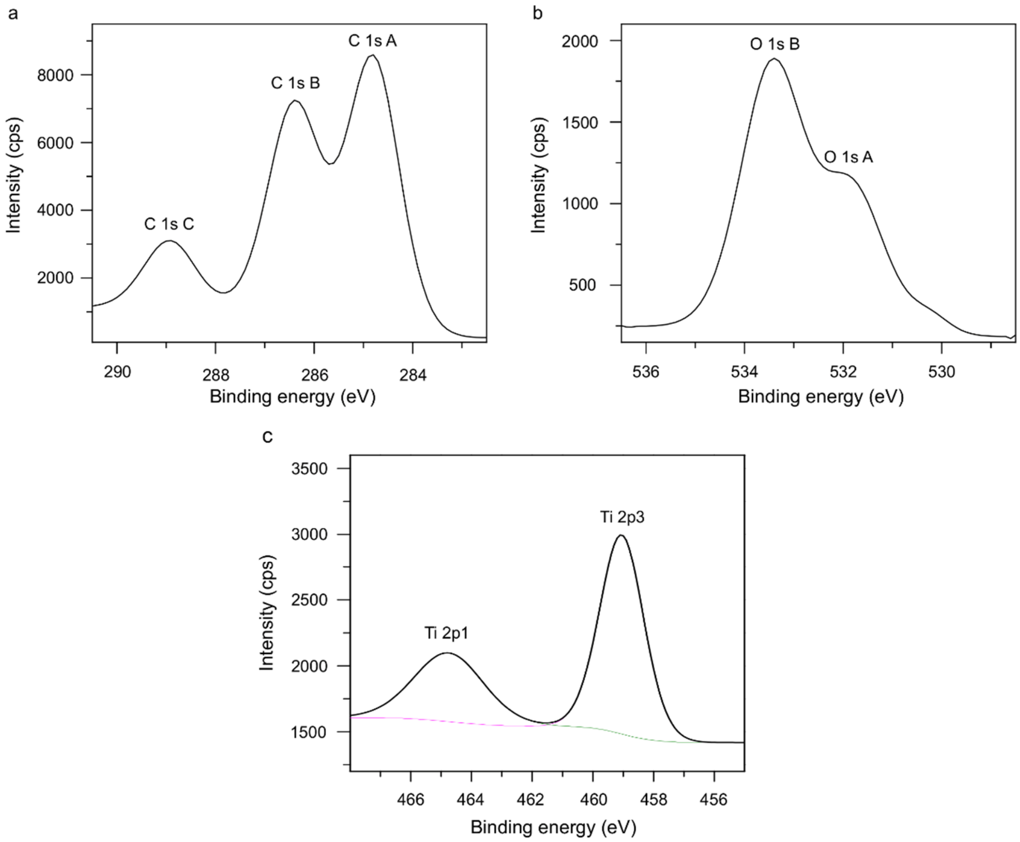

Figure S8. The XPS spectra of the TA–Ti^{4+} coating. (a) C1s peaks at ~284.7, ~286.4, and ~288.8 eV and (b) O1s peaks at ~532.0 and ~533.3 eV originating from TA in the coating. (c) Ti 2p peaks at ~459 and ~465 eV showing the presence of Ti^{4+} ions as crosslinkers in the coating.

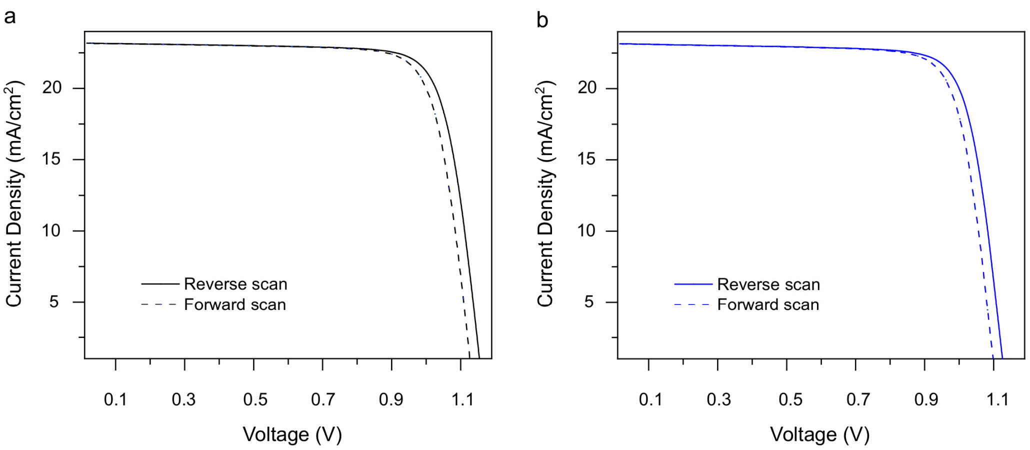

Figure S9. J-V plots of the (a) control and (b) encapsulated PSCs showing both forward and reverse scan characteristics. The hysteresis initially is the devices does not change with addition of the encapsulant film.

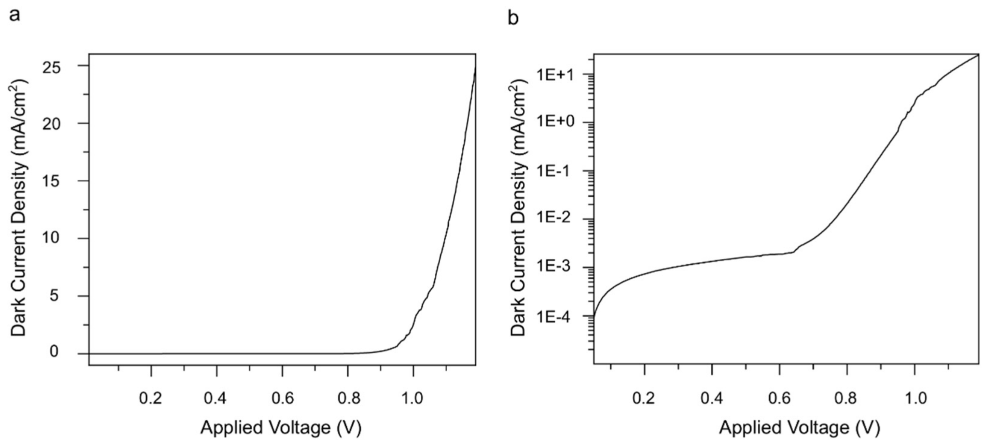

Figure S10. The dark current characteristics of the encapsulated cells are shown in (a) linear and (b) log scale. The linear plot provides a good approximation of the V_{OC}, and different regions of operation of the device as observed from the log scale plot.

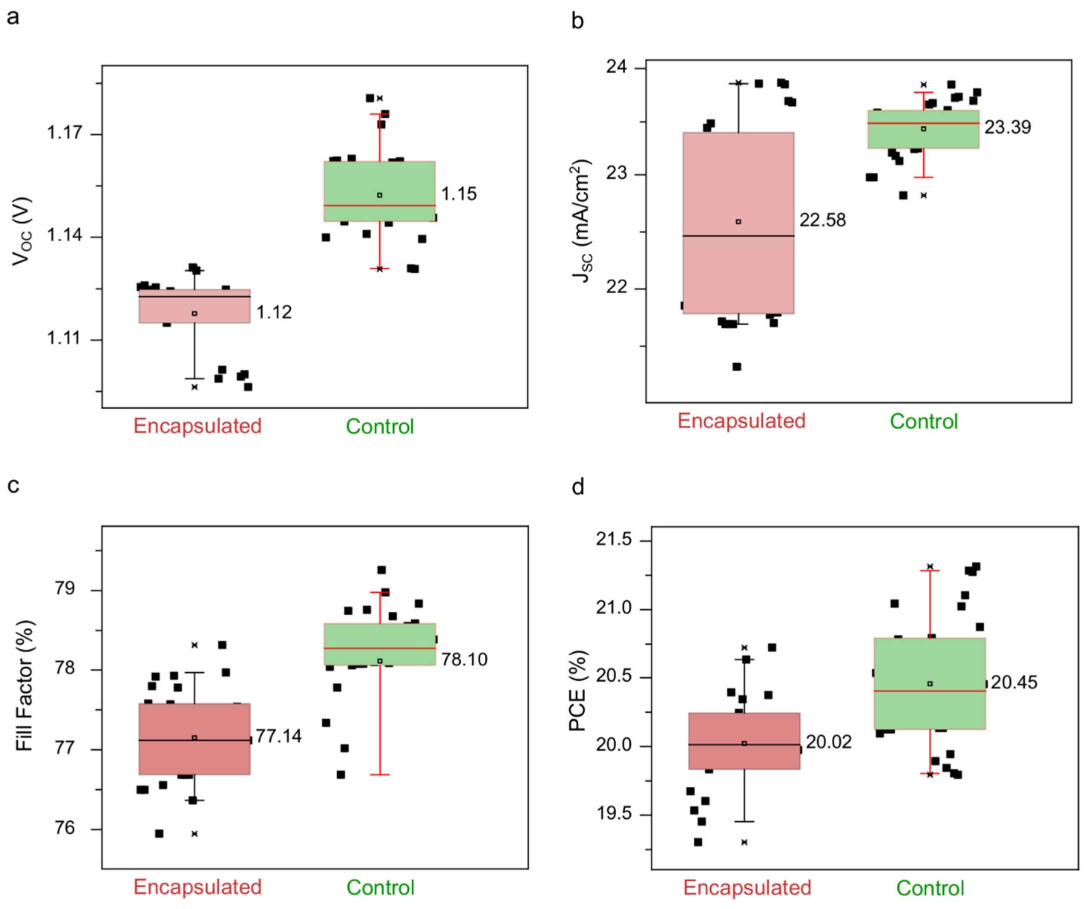

Figure S11. The statistical distribution of photovoltaic characteristics (a) V_{OC}, (b) J_{SC}, (c) FF, and (d) PCE of the cells with and without encapsulation. The data were taken from 25 cells for each type. All data were taken the next day after preparing the devices, stored in an N_2 filled environment.

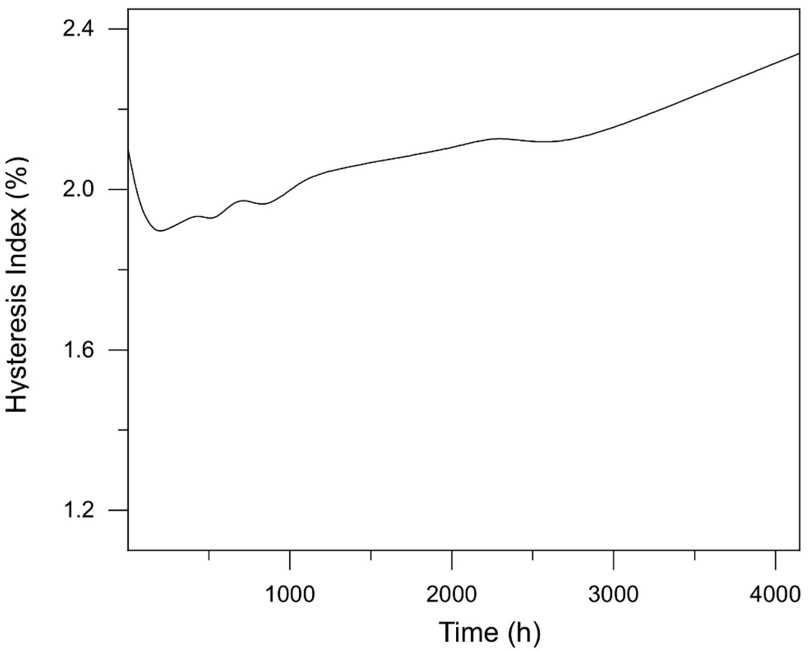

Figure S12. The time-dependent hysteresis index of the encapsulated PSCs showing that the PCE does not alter significantly as the sweep direction was changed even after ~4000 hours.

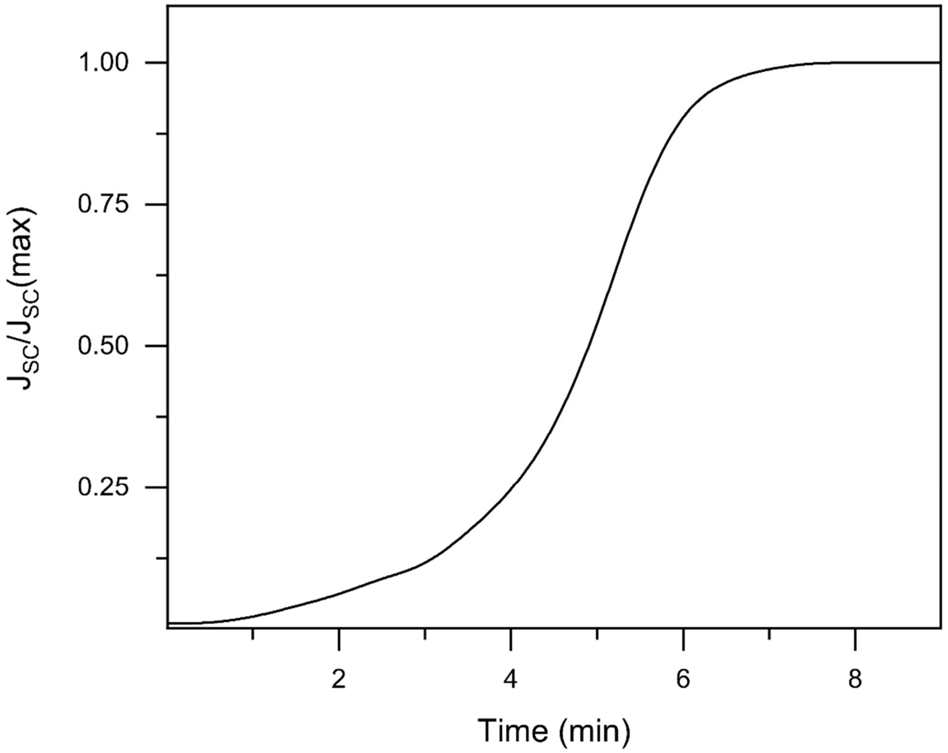

Figure S13. The current built-up in the encapsulated devices during first I-V measurement.

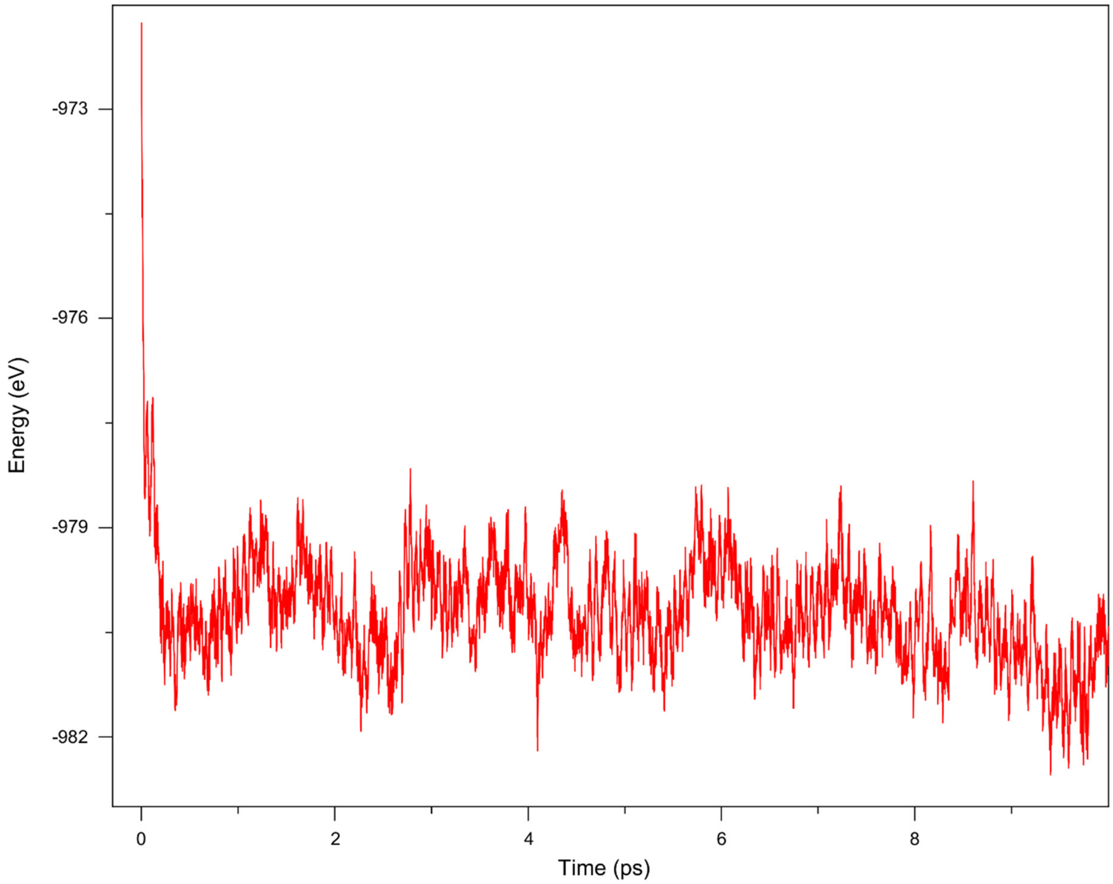

Figure S14. Total energy of the system as a function of time in our molecular dynamics simulation.

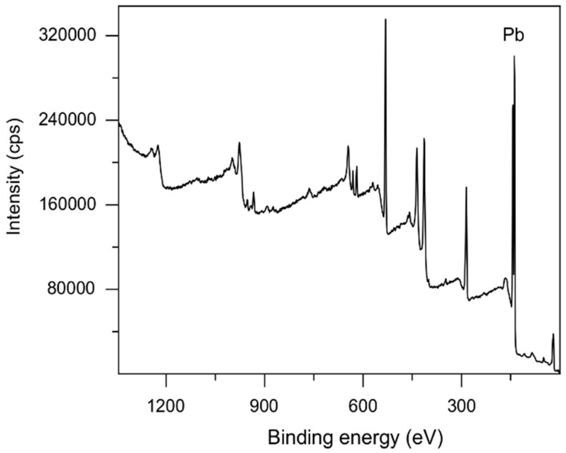

Figure S15. XPS survey spectra of the TA–Ti^{4+} coating after immersing in Pb^{2+} solution for 24 hours.

Table S1. Different PCE Lifetime of the control and Encapsulated cells

Lifetime	Champion Control (h)	Average Control (h)	Champion Encapsulated (h)	Average Encapsulated (h)
T90	45	42.4	650	631.5
T85	56	51.9	1400	1353.3
T80	66	63.1	2050	1959.2
T75	74	69.7	3600	3470.8
T70	87	81.4	7050	6883.5

Table S2. Fitted decay parameters for the time-resolved photoluminescence (TrPL) generated using the bi-exponential model in Decay Fit

Device / Age		A_1	A_2	τ_1 (ns)	τ_2 (ns)
0 h	Control	82.5	17.5	2.5	371.6
	Encapsulated	84.9	15.1	2.4	365.0
500 h	Control	92.7	7.3	1.7	260.5
	Encapsulated	86.2	13.8	2.2	339.1
3200 h	Control	99.8	0.2	0.3	143.9
	Encapsulated	91.4	8.6	1.8	279.4

References:

[1] J. R. Lakowicz, *Principles of fluorescence spectroscopy* **2006**, Springer.

[2] H. J. Snaith, A. Abate, J. M. Ball, G. E. Eperon, T. Leijtens, N. K. Noel, S. D. Stranks, J. T.-W. Wang, K. Wojciechowski, W. Zhang, *J. Phys. Chem. Lett.* **2014**, 5, 1511-1515.

[3] Y. Rong, Y. Hu, S. Ravishankar, H. Liu, X. Hou, Y. Sheng, A. Mei, Q. Wang, D. Li, M. Xu, J. Bisquert, H. Han, *Energy Environ. Sci.* **2017**, 10, 2383-2391.

[4] S. N. Habisreutinger, N. K. Noel, H. J. Snaith, *ACS Energy Lett.* **2018**, 3, 2472-2476.

[5] G. Kresse, J. Furthmüller, *Phys. Rev. B* **1996**, 54, 11169-11186.

[6] G. Kresse, J. Furthmüller, *Comput. Mater. Sci.* **1996***,* 6, 15-50.

[7] G. Kresse, J. Hafner, *Phys. Rev. B* **1994**, 49, 14251-14269.

[8] G. Kresse, J. Hafner, *Phys. Rev. B* **1993**, 47, 558-561.

[9] G. Kresse, D. Joubert, *Phys. Rev. B* **1999**, 59, 1758-1775.

[10] P. E. Blochl, *Phys. Rev. B* **1994**, 50, 17953-17979.
